# Sydenham Society

**Published:** 1845-06

**Authors:** 


Sydenham Society. *Observations on Aneurism, selected from the Works of the Principal writers on the Disease, from the earliest periods to the close of the last century.* Translated and edited by John E. Erichsen, Lecturer on General Anatomy and Physiology, at the Westminster Hospital. 8vo., pp. 524 ; London, 1844.

These are the two first publications of the Sydenham Society for the year 1844—the work of Paulus of Ægina being itself fully worth the whole year's subscription. Were it, indeed, published in the ordinary manner by booksellers, it could scarcely be afforded for *five dollars.* Yet, for this trifling sum, three elegant volumes, beautifully got up, and learnedly edited, are furnished to the members or subscribers. Were the advantages of the Society more fully known, it could not fail to be most extensively patronised. Already, indeed, its members amount to about 2000, which places at the disposal of the Council the sum of 2000 guineas annually, or upwards of 10,000 dollars, to be expended on the publication of such works as they may deem most advisable, consistently with the objects for which the Society was instituted.

Although it is highly interesting to be well acquainted with the productions of so distinguished a writer as Paulus, the volume before us—as its title indicates—is not confined to a mere exposition of his opinions. Its great value, indeed, consists in its containing a learned commentary by Mr. Adams on the knowledge possessed by the ancients of the various subjects canvassed by Paulus; and hence it becomes a valuable book of reference upon numerous topics of medical history.

"I am sensible," says Mr. Adams, "that it is to the growing conviction in the profession of the value of ancient authorities, that I owe the very flattering distinction which my work has now obtained; and I shall have great satisfaction in reverting to the labours of former years, if they should now prove instrumental in increasing the desire of becoming acquainted with the views and practice of our forefathers. That the ancient literature of medicine has been too much neglected in this country is not disputed by any competent judge; and it would appear from the remarks of M. Hecker, in his address to the physicians of Germany, given in his admirable work on Epidemics, (Dr. Babington's Translation; Sydenham edition, p. 15,) that the profession on the continent is not much in advance of us in this respect." p. vi.

It may be remarked, that in the year 1834 Mr. Adams published the first three books of Paulus, with a commentary; but, "notwithstanding the very favourable reception which that volume obtained from many of the most eminent members of the medical profession, as well as from scholars, both at home and abroad," he was under the necessity of deviating from his original intention of completing the production of the work in the same form, and at his own risk; and it is one of the characteristic merits of the Sydenham Society, that it undertakes works of eminent worth and rare interest, which would, notwithstanding these qualities, prove unprofitable as mercantile speculations, to both author and publisher. In regard to ancient medical literature, the idea is too prevalent that it merely instructs us in opinions which have long passed away, and that its study leads to no practical information. The *cui bono* is ever a miserable argument; but in the present case more than usually so; for, as Mr. Adams has remarked, "what renders ancient medical literature of the more importance at the present day, is the circumstance that it is almost our only source of information, with regard to the diseases prevalent in several extensive countries bordering upon the Mediterranean Sea. It is well known that the inhabitants of Greece, of Asia Minor, and of the north coast of Africa, have been long sunk into such a state of intellectual decrepitudé, as renders them incapable of making and recording original observations; consequently, for information in regard to the phenomena of disease, as manifested in these regions, we are almost entirely thrown back upon the literature of their ancestors." p. vii.

Two more volumes will be required to complete the work.

The volume on Aneurisms is "got up" in the same admirable style as its precursors, but we doubt whether the selection will give as much, or as general, satisfaction as they did. It is but comparatively of late years, that the pathology and therapeutics of aneurismal affections have been well comprehended, and it is little more than a matter of curiosity for us to inquire into the opinions formerly indulged in on those subjects. Certainly our therapeutical knowledge cannot be augmented by it. Mr. Erichsen has given extracts from systematic works, commencing with those of Galen, and the first memoir selected bears the date of 1681. The selections are not brought down later than the close of the last century, "as it was thought, that most works of any importance, that have appeared since then, would probably be either in the possession, or within the reach of the greater number of the members of the Society; and, moreover, that it was desirable to avoid the invidious and delicate task of making extracts from, or of reprinting, the works of living authors. The Bibliography contains a reference to all memoirs and detached essays of any value, that have been published on the subject of aneurism within the period to which the work is confined. Those detached cases, also, that are of more than usual interest, are included in it. The volume is divided into three parts. The *first* containing those memoirs and extracts that relate more particularly to the symptoms, causes, and pathology of aneurism; the *second*, those that refer to the treatment of this disease,—the materials of each of these parts being arranged chronologically: the *third* part consists of the Bibliography.

We again strongly recommend the Sydenham Society to the attention of our readers;—reminding them, that not one of the works published by the Society can be obtained except by *subscribers;* and that the number of copies annually printed is regulated by the number who have paid the contribution of five dollars. The volumes issued in the first year, are now out of print, and happy may he feel who was fortunate enough to subscribe at the commencement.

